# Puzzle-based versus traditional lecture: comparing the effects of pedagogy on academic performance in an undergraduate human anatomy and physiology II lab

**DOI:** 10.1186/s12909-015-0390-6

**Published:** 2015-06-23

**Authors:** Lucas Stetzik, Anthony Deeter, Jamie Parker, Christine Yukech

**Affiliations:** University of Akron, Akron, OH USA

**Keywords:** Puzzle, Lecture, Teaching, Pedagogy, Anatomy, Physiology, Performance, Concept

## Abstract

**Background:**

A traditional lecture-based pedagogy conveys information and content while lacking sufficient development of critical thinking skills and problem solving. A puzzle-based pedagogy creates a broader contextual framework, and fosters critical thinking as well as logical reasoning skills that can then be used to improve a student’s performance on content specific assessments. This paper describes a pedagogical comparison of traditional lecture-based teaching and puzzle-based teaching in a Human Anatomy and Physiology II Lab.

**Methods:**

Using a single subject/cross-over design half of the students from seven sections of the course were taught using one type of pedagogy for the first half of the semester, and then taught with a different pedagogy for the second half of the semester. The other half of the students were taught the same material but with the order of the pedagogies reversed. Students’ performance on quizzes and exams specific to the course, and in-class assignments specific to this study were assessed for: learning outcomes (the ability to form the correct conclusion or recall specific information), and authentic academic performance as described by (Am J Educ 104:280–312, 1996).

**Results:**

Our findings suggest a significant improvement in students’ performance on standard course specific assessments using a puzzle-based pedagogy versus a traditional lecture-based teaching style. Quiz and test scores for students improved by 2.1 and 0.4 % respectively in the puzzle-based pedagogy, versus the traditional lecture-based teaching. Additionally, the assessments of authentic academic performance may only effectively measure a broader conceptual understanding in a limited set of contexts, and not in the context of a Human Anatomy and Physiology II Lab.

**Conclusion:**

In conclusion, a puzzle-based pedagogy, when compared to traditional lecture-based teaching, can effectively enhance the performance of students on standard course specific assessments, even when the assessments only test a limited conceptual understanding of the material.

## Background

Throughout the past 25 years, studies involving teaching methods that differ from the standard lecture/memorization/testing format have shown promise in increasing the level of authentic intellectual performance demonstrated by students [1–5]. Thus, in more recent studies it has been argued that traditional teaching methods convey information and content while lacking sufficient development of critical thinking skills and problem solving [6]. The framework used to score non-traditional teaching methods has focused on learning outcomes, and content-specific conceptual understanding [6, 7]. In an effort to assess the broader effects of pedagogy on a student’s critical thinking skills, this study has implemented a framework with which to determine the level of students’ authentic intellectual performance [8–13]. The authentic intellectual performance framework used for this study was based on the following standards; higher-order thinking, depth of knowledge, connectedness to the world beyond the classroom, substantive conversation, and social support for student achievement.

Previous studies have established definitions for project-, problem-, and puzzle-based teaching methods in which each method builds upon those before it [6]. The least abstract method, project-based, includes working in teams and dealing with uncertainty and changing conditions. Built upon this method is problem-based learning; this method involves acquiring domain-specific knowledge and reasoning with domain-specific methods. The most abstract method, puzzle-learning, builds upon both of these to develop critical thinking and logical reasoning independent of a specific domain [6, 14–17]. The first two methods have been studied extensively, but little has been done with regards to puzzle-based learning [18, 19].

Our research aims to assess the impact of puzzle-based learning on conventionally valued academic knowledge such as: course specific material and course specific conceptual understanding, as well as broadly valued skills, such as conceptual reasoning independent of course material. Non-traditional lecture, memorization, and testing methods enable in-class activities to engage students in a wide range of intellectual skill sets both conventionally academic and broadly applicable [20–22]. In-class activities that use puzzle-based teaching can require students to connect to their prior knowledge, explain, interpret, and apply newly acquired knowledge, as well as develop personal perspective and understanding [4, 5].

The objective of this research was to compare the effects of traditional teaching methods and puzzle-based teaching on authentic intellectual performance, and learning outcomes.

## Methods

**Fig. 1:**
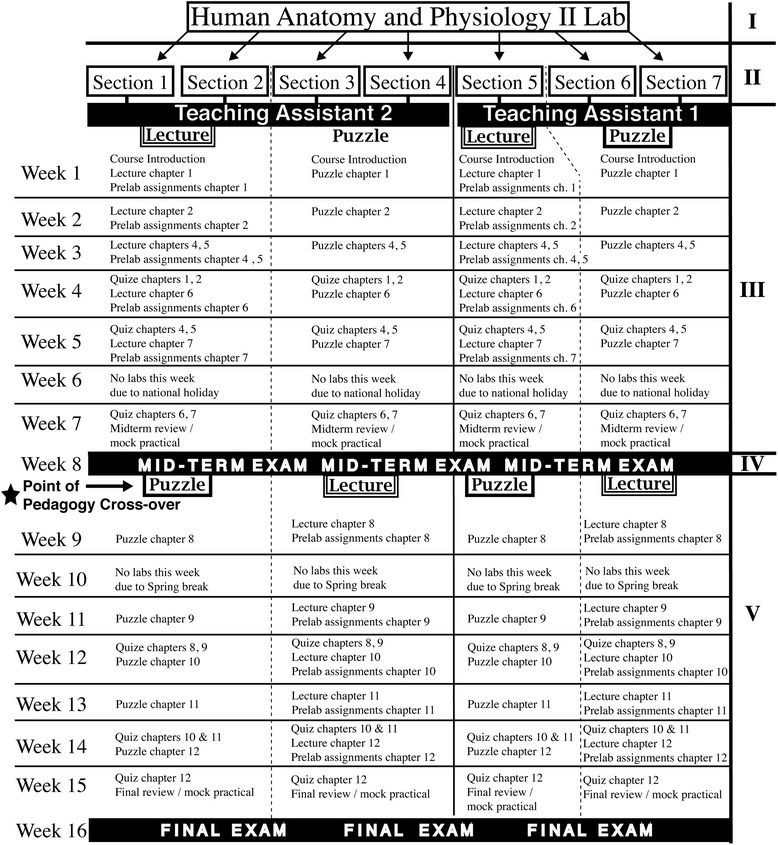
Course Structure and Single Subject/Cross-Over Design. A summary of the Human Anatomy and Physiology II Lab course schedule including the implementation of a single subject/cross-over design. I) Course offered at the University of Akron, II) Individual sections or “classes” each approximately 25–30 undergraduate students (185 in total). Each section was assigned to meet at the same unique specified time and classroom on campus at the University of Akron. The actual meeting times, and section numbers have been omitted to ensure anonymity of the participants in keeping with the requirements set forth by the Internal Review Board at the University of Akron. III) Weeks 1–8 of an academic semester, including indications of how sections were divided among the teaching assistants, and which sections began the study with either a traditional lecture or puzzle-based pedagogy. IV) The mid-term exam and, as indicated by the black five pointed star, the point of pedagogy cross-over. V) Weeks 9–16 of an academic semester, including indications of which sections crossed-over to either a traditional lecture or puzzle-based pedagogy

The Internal Review Board at the University of Akron deemed acquisition of all data involving human participants exempt from internal review board approval as the proposed methods did not pose a threat of harm to the participants, the methods were in keeping with standard educational practice, and the identities of each participant remained anonymous throughout the study. The participants chosen for this research were students taking the Human Anatomy and Physiology II Lab at the University of Akron. Seven sections of this lab, 185 students in total, were taught using standard teaching practices as well as puzzle-based methods, and their level of authentic intellectual performance was measured in addition to their standard course assessments. Two teaching assistants were utilized among seven sections of the course (Fig. 1).

Human Anatomy and Physiology II Lab is a prerequisite for all medical related degrees including but not limited to: nursing, pre-med, exercise science, biochemistry, and biology at the University of Akron. The proportion of students representing these specific programs was not controlled, but the intended majors of each student were documented in case there was a strong program related bias observed in any of the six sections. Such a bias was not apparent in the results and so this was not included as a factor in the final analysis.

### Single subject/cross-over

As a condition for conducting this study in the Human Anatomy and Physiology II Lab, it was required by the University of Akron that all the course material be presented in a specific order. This order is designed such that the material prior to the mid-term exam is more challenging than the material after the mid-term exam. Consequently, there could be a bias toward improved scores on standard course assessments in the second half of the semester independent of any manipulations to teaching-style (*i.e.* scores on the final exam may be higher than the mid-term exam).

Knowing that this bias could effect the interpretation of our results by showing improvement in any teaching style used in the second half of the semester, a single subject/cross-over design was implemented (Fig. 1) to compensate for this bias. Each teaching assistant taught two sections of the course using a puzzle-based pedagogy for the first half of the semester. After the midterm practical exam, these sections then switched to a traditional lecture-based pedagogy for the remainder of the semester. The remaining sections started with a lecture-based pedagogy and then moved to a puzzle-based pedagogy after the midterm practical exam.

By using the single subject/cross-over design, it can be determined if the order in which the teaching style was used has a significant effect on student performance. Such significance would potentially confirm the suspected bias towards improved scores on standard course assessments in the second half of the semester, and would be the result of a decreased difficulty in the course material. Additionally, by using this design, it can be determined if teaching-style, independent of the order in which the teaching-style was used, had a significant effect on student performance. Taken together, this means that if both order and teaching-style are significant that there is a bias towards improved scores in the second half of the semester, and that teaching-style did have a significant effect on student performance independent of this bias.

### Traditional/lecture design

**Fig. 2:**
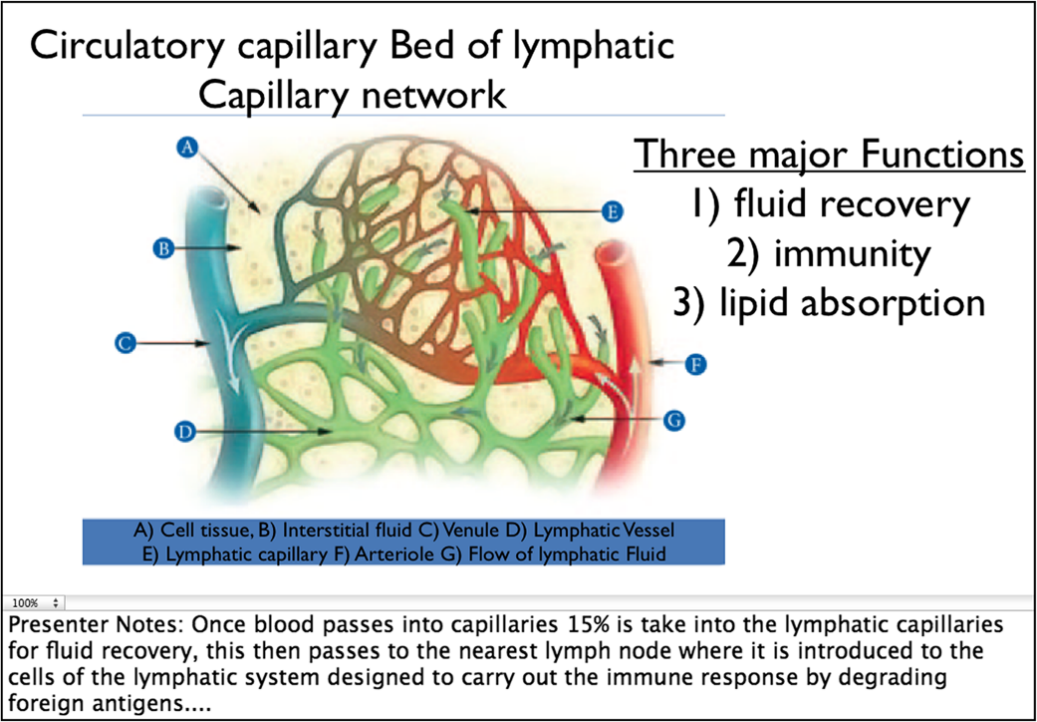
Presentation Slide For Traditional Lecture-Based Pedagogy with Presenter Notes. An example PowerPoint slide describing course material from chapter 7 of the Human Anatomy and Physiology II Lab complete with presented notes used during the traditional lecture-based pedagogy portion of the study

**Fig. 3:**
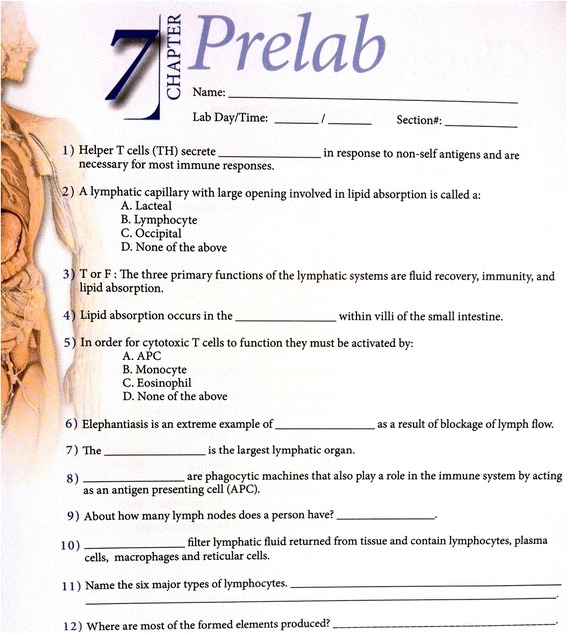
In-class Prelab Assignment For Traditional Lecture-Based Pedagogy. The prelab activity for chapter 7 of the Human Anatomy and Physiology II Lab, for use in the traditional teaching portion of the class

The traditional lecture-based portion of the course used a projector screen and Microsoft Power Point to display images and key information related to the week’s lab. The teaching assistant read through a prepared set of notes related to the content of the chapter, as they displayed the slide show. Figure 2 is an example of a Power Point slide and some of the notes that were presented to the class. Once the lecture was over, the students had the remainder of the class period to complete a prelab activity (Fig. 3) usually consisting of approximately twelve fill-in-the-blank questions, using their lab manual and the models in the classroom.

The students were asked to briefly describe any additional applications of the concepts learned in that week’s activity, as a measure of authentic intellectual performance. Every picture in the lab manual had one or more models of the same anatomical structure physically present and available during the class period. At the end of the class period, each student’s prelab was graded for correctness and returned to the student so that they could study from it in the following week.

### Puzzle design

#### Content specificity, and conceptual generality

As previously stated, a key feature of puzzle-based learning that distinguishes it from problem-based or project-based learning is the emphasis on domain-independent critical thinking and abstract reasoning [6, 16, 17]. This is not to say that a puzzle-based learning course is about presenting and discussing a variety of puzzles, but rather that it is about presenting and understanding problem-solving principles through puzzles that serve both as an illustration of broad concepts and of course specific material [6, 17].

This study’s puzzle design used the Human Anatomy and Physiology II Lab course content as a vehicle for the development of domain-neutral basic reasoning skills. In general, the participants would have to familiarize themselves with a set of “rules” given in the form of background information regarding a real-world medical application of the course material.

For example if the chapter material focused on the lymphatic system, a real-world medical application for the lymphatic system was the diagnosis of lymphoma (a cancer specific to the lymphatic system) through histological test results. As previously mentioned, the “rules” for this puzzle were not directly stated; the participant would come to know them by a conceptual understanding of the chapter material in the lab manual, and any supplementary material provided in the form of a photocopied handout on the day of class. In the case of the lymphoma puzzle, such an understanding should have yielded the following rules:

1. The cancer will only affect anatomical structures specific to the lymphatic system.
2. The lymphatic system has a particular directional flow by which the cancer can spread.
3. When viewed with basic staining techniques, some types of lymphatic cancer cells have one distinctive morphology, are highly metastatic, and thus have the ability to spread throughout major anatomical features, while other types of cancer cells have a different type of morphology and are only able to spread through minor substructures of the lymphatic system.

While this may appear to be a domain specific problem, the puzzle “rules” directed the participants to develop a set of basic reasoning skills independent of the chapter content. Stated more abstractly, the rules could be simplified to:

1. This is a closed system, the limits of which are defined.
2. This closed system has sequential order through which it functions on two size dependent scales.
3. Idea 1 affects the system on one size scale; Idea 2 affects the system on a different size scale. Idea 1 and 2 are mutually exclusive.

In-addition to learning critical thinking and basic reasoning skills, a puzzle-based in-class activity reinforces the specific chapter material by putting it into a contextual framework. Where a traditional lecture would state the directional flow of the lymphatic system on two size scales, its various anatomical structures, and its physiological relevance, a puzzle-based in-class activity reinforces that same information by making it impossible to complete the assignment without a conceptual understanding of the material.

#### Assessments of authentic intellectual performance (AIP)

At the end of the puzzle activities and the prelab activities, participants were asked to identify any additional applications of the concepts from that activity’s chapter material. This provided a means through which to assess the participants’ broader conceptual understanding of the material. This broader conceptual understanding should, in theory, translate to “real-world” aptitude [9–12]. These assessments were scored using the authentic intellectual performance criteria outlined by Newmann and Wehlage [10]. These standards were developed in order to represent the quality of intellectual work without being linked to any one teaching method [8, 13, 10]. Each of the following standards; higher-order thinking, depth of knowledge, connectedness to the world beyond the classroom, substantive conversation, and social support for student achievement; was designed as a continuous, analog representation of quality.

In general, scores were given on a scale of 0–5 in which answers that demonstrated little conceptual understand beyond the domain specific context of the puzzle material were given a 1–2, answers that demonstrated conceptual understanding beyond the domain specific content but lacked an application of the concept outside of the domain specific content received a 3, answers that demonstrated a conceptual understanding beyond the domain specific content and an application of the concept that was beyond the domain specific content of the puzzle but were still within the domain specific content of the course received a 4, and answers that demonstrated a conceptual understanding beyond the domain specific content and an application of the concept that was beyond the domain specific content of the puzzle and the course received a 5.

For example, in the puzzle about the lymphatic system, the participants provided some variation of the following answers and received the following scores:

*The Lymphatic system has lymph nodes all over the body that move lymphatic fluid and nutrients in the cells.* (Score 1 or 2)

*The lymphatic system is a mechanism for biological cleaning and nutrient transport*. (Score 3)

*The lymphatic system moves waste out of the cell and moves nutrients into the cell, much the way the act of eating and digestion moves nutrients into the body and waste out of the body* (score 4)

*The lymphatic system is like the water treatment center for a city, it moves clean fluid into homes and keeps the residents alive, similar to how the lymphocytes keep cells of the tissue alive, and then the pipes connected to the sewage system remove waste and keep people in their homes clean, the way lymphatic fluid removes wastes from cells.* (Score 5)

If the student failed to answer the question they received no credit.

One important feature of this assessment is that it demonstrates a broader conceptual understanding independent of the puzzle content [10]. We assume that this broader understanding would be difficult to achieve without the use of basic reasoning. This assumption implies that if a puzzle/project/problem requires the participant to use and develop basic reasoning, this basic reasoning will facilitate the connection of domain specific concepts to domain independent concepts [6, 16, 17].

#### Standard course assessments

On the days the class had a quiz, the quiz tested a subset of course material defined by the course syllabus, and then the day’s activity began once all the quizzes were collected by the teaching assistant. The quiz format was specific to the structure of the Human Anatomy and Physiology II Lab course, prior to the inclusion of this study. Students were presented with a Power Point slideshow in which each slide had one of the figures from the student’s lab manual with its structures labeled by an alphabetic letter. The teaching assistant then informed the class which letters needed to be identified as anatomical structures, or asked a question relating to physiological function and included what number-line they should write the anatomy term next to on their quiz sheets. The assessments had a total of 30 questions, and in general 5–8 of these questions would be related to a physiological concept while the remainder of the questions were anatomical identification.

The mid-term and final lab practical exams were also a format specific to the structure of the Human Anatomy and Physiology II Lab course, prior to the inclusion of this study. In these assessments, a total of 20 anatomical models or histological images were assigned individual stations throughout the exam room. Students completed a total of 2, 1-min rotations for each station in sequential order. Each station had 2–3 questions that either required the student to identify a particular anatomical structure indicated by a numbered sticker on the model or image, or to answer a question about physiological function related to that station’s model or image. The assessments had a total of 50 questions, 30 of which were anatomical identification questions, and 20 of which were physiological function questions.

All standard course assessments were graded for correctness. To receive full credit, the answers needed to be complete and all vocabulary terms needed to be spelled correctly. Incorrect spellings different by a single vowel received ¾ credit; incorrect spellings by a nonessential consonant (C instead of K or S, *etc.*) received ½ credit. All other misspellings received no credit.

#### Statistical analysis

Analyses were done with IBM SPSS Statistics version 21. Multivariate analysis of variance (MANOVA) was used to determine if there were between-subjects effects of pedagogical styles on the scores of standard course assessments. MANOVA was used because this method can analyze significant differences in student performance on standard course assessments as single factor, effectively combining the tests and quizzes to look for an overall effect with regards to teaching-style, order, and teaching-style + order (Table 1). A similar MANOVA analysis that separated the standard assessments into quizzes and tests was used because this method can analyze significant differences in student performance on tests and quizzes separately to look for an effect with regards to teaching-style, order, teaching-style + order (Table 2). Additionally, this analysis was split by TA to determine the effect of subtle difference in instruction on teaching-style, order, and teaching-style + order (Table 3). For the AIP assessments a univariate analysis of variance was used to determine between-subjects effects with regards to teaching-style, order, and teaching-style + order. For all analyses results were considered statistically significant when *p* < 0.05.

**Table 1:** MANOVA comparing scores on standard assessments between pedagogies

Effect	Wilks’ Lambda	F	Hypothesis df	Error df	*p*-value
Teaching Style	.95	5.588^a^	3.000	298.000	0.001*
Order	.88	13.459^a^	3.000	298.000	0.000*
Teaching Style + Order	.83	16.353^a^	3.000	298.000	0.000*

*significant at *p* < 0.05

^a^ exact statistic

**Table 2:** MANOVA comparing scores on standard assessments between pedagogies with tests for between-subjects effects on quizzes and tests

Multivariate tests					Between subject effects	
Effect	Wilks’ Lambda	F	Hypothesis df	Error df	Dependent variable	*p*-value
Teaching style	.95	5.588^a^	3.000	298.000	Quizzes	0.876
					Tests	0.846
Order	.88	13.459^a^	3.000	298.000	Quizzes	0.006*
					Tests	0.001*
Teaching Style + Order	.829	16.353^a^	3.000	298.000	Quizzes	0.000*
					Tests	0.186

*significant at *p* < 0.05

^a^ exact statistic

**Table 3:** MANOVA comparing scores on standard assessments between pedagogies for ta1 and ta2 with tests for between subject effects on quizzes and tests

Multivariate tests											Between subject effects		
Effect	Wilks’ Lambda		F		Hypothesis df		Error df		p-value		Dependent variable	*p*-value	
TA	TA1	TA2	TA1	TA2	TA1	TA2	TA1	TA2	TA1	TA2		TA1	TA2
Teaching style	.98	.97	1.421^a^	3.016^a^	2	2	167	183	.244	.005*	Quizzes	.107	.758
											Tests	.702	.041*
Order	.96	.86	.959^a^	15.024^a^	2	2	167	183	.030*	.000*	Quizzes	.205	.000*
											Tests	.159	.000*
Teaching Style + Order	.90	.85	.902^a^	15.698^a^	2	2	167	183	.000*	.000*	Quizzes	.000*	.205
											Tests	.068	.000*

*significant at *p* < 0.05

^a^ exact statistic

It is important to note that the significant effect of order, and teaching-style + order demonstrated a statistically significant improvement in student performance on standard course assessments in the second half of the semester (data not shown). However, the single subject/cross-over design of the study removes this bias when analyzing the effect of teaching style exclusively.

## Results

**Fig. 4:**
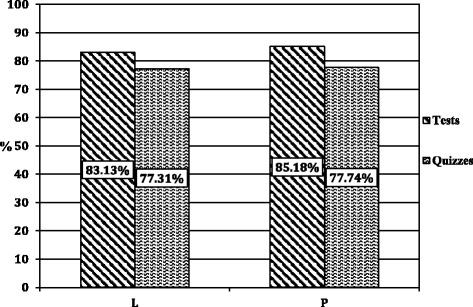
Standard Course Assessments. Mean scores for standard assesments. Both tests and quizzes showing a significant inprovement (*p < 0.05*) in the puzzle-based pedagogy (P) versus traditional lecture-based teaching (L). Tests are an average of final and mid-term exams. *N = 185*

**Fig. 5:**
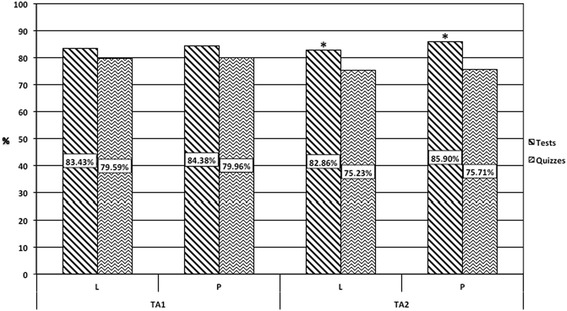
Standard Course Assessments Separated by Tests, Quizzes, and TA. Mean scores for standard assesments. Tests for TA2 showing a significant inprovement (*p < 0.05*) in the puzzle-based pedagogy (P) versus traditional lecture-based teaching (L). Tests are an average of final and mid-term exams. TA1 *N = 97,* TA2 *N = 88*

Overall, our findings indicate a significant improvement in students’ performance on standard course specific assessments using a puzzle-based pedagogy versus a traditional lecture-based teaching style. Quiz and test scores for students improved by 2.1 and 0.4 % respectively in the puzzle-based pedagogy, versus the traditional lecture-based teaching. Mean quiz and test scores for puzzle-based students were 85.2 and 77.7 respectively, while the mean quiz and test scores for traditional lecture-based teaching were 83.1 and 77.3 respectively (Fig. 4). The significance of teaching-style independent of order in combination with the cross-over design suggests that the significant bias of order was effectively compensated for (Table 1). If the bias of order were not effectively compensated for there would be no significance in teaching-style independent of order as scores on all standard assessments increased in the second half of the semester independent of teaching-style. Interestingly when analyzing the effect of teaching-style on the specific outcomes of tests and quizzes separately neither is significant (Table 2). However, when these results are further analyzed for the effect of teaching-style on the specific outcomes of test and quizzes with respect to individual TAs the test scores for TA2 remain significantly improved by 3.04 % (Fig. 5, Table 3).

**Fig. 6:**
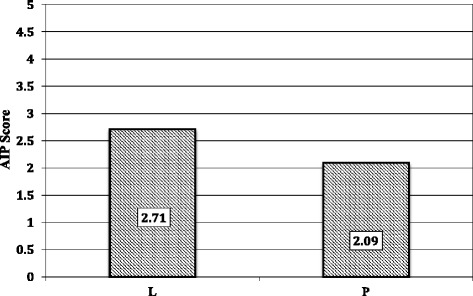
Assessment Authentic Intellectual Performance. Mean scores for AIP assessments showing a significant difference (*p < 0.05)* between the puzzle-based pedagogy (P) and traditional lecture-based teaching (L). *N = 185*

Additionally, in terms of the assessments for authentic intellectual performance (AIP), our findings indicate a significant difference (*p* = 0.001) between puzzle-based and traditional lecture-based pedagogies (Fig. 6). However, based on anecdotal accounts of interactions between the teaching assistants and participants, we also report that these assessments contained a number of complicating factors that may have contributed to a fundamental inaccuracy of the significance in this study, and failure to assess the participant’s potential for broader reasoning and conceptual understanding independent of domain-specific content.

## Discussion

Taken together, the results of the assessments for learning outcomes and authentic intellectual performance help to inform the relative effectiveness of puzzle-based pedagogies as a tool for “open” or “experiential” learning theories in comparison with more didactic theories, the primary vehicle of which is a traditional lecture based pedagogy [8]. Previous studies have demonstrated the effectiveness of implementing “open/experiential” learning through a puzzle-based pedagogy in mathematics and computer science courses [6, 16, 17]. This study expands the academic context of “open/experiential” learning theories into a Human Anatomy and Physiology II Lab that is traditionally assumed to be rooted in a didactic theory, through dry anatomical identification and rote memorization. By moving “open/experiential” learning into this new academic territory it could be possible, as previous studies have suggested, that educational techniques such as puzzle-based and problem-based pedagogies are effective in a broad range of academic contexts [8]. The findings of this study suggest a significant improvement in performance with puzzle-based versus traditional lecture-based pedagogy, on standard Human Anatomy and Physiology II Lab assessments. Additionally, there is an arguably unreliable significance in assessments of authentic intellectual performance.

One explanation for the significant improvement in performance on the standard assessments between puzzle-based and traditional lecture-based pedagogies is that the puzzle-based pedagogy creates a contextual framework for the course material that improves students’ ability to recall specific details. Previous studies have demonstrated the increase in student performance using a puzzle-based pedagogy in advanced mathematics courses and computer science courses [6, 16, 17]. The assessments for these courses require students to have a conceptual understanding of the material in order to grasp challenging abstract concepts.

The standard assessments for a human anatomy and physiology lab, however, are ~80 % anatomical identification and ~20 % physiology. Neither set of questions in the Human Anatomy and Physiology II Lab *requires* a deeper conceptual understanding of the material in order to perform well on the standard assessments. Rather, the assessments are geared toward rote memorization, devoid of conceptual understanding. Despite the vast differences between Human Anatomy and Physiology II Lab and the previous studies that have implemented puzzle-based learning in advanced mathematics and computer science courses [6, 16, 17] in terms of course design and assessments, the contextual framework established by the puzzle-based pedagogy seems to aid in the recall of specific details improving student performance when compared to traditional lecture-based teaching.

### Importance of individual characteristics

The significance of pedagogy in teaching assistant #1’s sections presents a very important observation that is intuitively obvious and methodologically difficult to demonstrate. This observation being that the individual characteristics of the teaching assistant significantly impacts the performance of students. In this study the individual characteristics that may have affected students’ performance are: familiarity with the course material, approachability, and style of fielding questions during class activities.

While neither teaching assistant had presented an in-class lecture or a puzzle activity for this course, Teaching assistant #2 (TA2) had previously taught this course once prior to this study. Additionally, because TA2 had prior experience with the material, their intellectual input was critical to the design of each in-class activity. Though both TAs met prior to instructing the chapter material for that week to ensure there was a uniformity in instruction, it is possible that TA2’s familiarity with that material may have influenced the ability answer activity related questions. Though both TAs did not directly provide any student with answers to any in-class activity, a greater familiarity with the material could possibly have give TA2 the ability to direct students into making conceptual connections more effectively. This is supported by anecdotally reported interactions between numerous participants and the lab coordinator/instructor, in which participants expressed a frustration and dissatisfaction with TA1’s level of preparedness and familiarity with in-class activates.

### Motivational effect of course expectation

The focus of Human Anatomy and Physiology Labs on anatomical identification and rote memorization is not only something that is commonly accepted and understood, but is expected by students enrolling in the course. Based on anecdotal reports of student-participant teaching assistant interactions, it was clear that the majority of students felt that the puzzle-based in-class activities were excessively difficult, and added little benefit to their understanding of the material. Many participants argued at great length with their teaching assistant about the puzzle activities and consistently expressed the view that Anatomy and Physiology was a memorization course. This predominantly negative attitude towards the puzzles contrasts the claims of many other studies that implement puzzle-based learning, and describe it as being “fun”, and possessing an “entertainment factor” [6, 17].

As previously mentioned, the course this study was conducted in was Human Anatomy and Physiology *II* Lab. Thus, it is important to note that all the participants had completed the Human Anatomy and Physiology *I* lab without any of the in-class puzzle activities used in this study. This generated a workload expectation that was exceeded by the in-class puzzle activities included in this study, and consequently an inherent motivational resistance to any potential benefit that could be gained through the in-class puzzle activities.

The most striking motivational resistance was apparent in the participants’ overwhelmingly negative attitude toward and effort applied to the AIP assessments. Specifically, the disproportional relation between the degree of perceived effort required to answer the assessment’s question correctly and the credit received for a correct answer. This resulted in many students applying little or no effort to the assessment and in many cases skipping it completely. This was especially true if the participant had applied a self-determined “great deal” of effort on an in-class assignment, and in most instances in which this occurred it was after completing a challenging puzzle activity. This is supported by the percentage of AIP assessments that received a score of “0” (a score of “0” was exclusively used to indicate that the question was left blank). The percentage of AIP assessments with a “0” in the puzzle-based portion of the study was 30.2 %, versus 14.5 % in the traditional lecture-based portion of the study. It is for this reason that we assume there exists a decrease in the students’ performance on this assessment during the puzzle-based portion of the study. It is possible that this trend in overall decreased performance in AIP scores over time is why other similar studies performed these assessments at only one time point during a semester (*i.e.* once in the spring and once in the fall) [10].

This may suggest that these assessments can only effectively measure a broader conceptual understanding in a limited set of contexts, and not in the context of a Human Anatomy and Physiology II Lab. Alternatively, this may suggest that in order to gain useful information using the AIP assessments that there must be fewer total assessments per individual, and/or that the assessments may need to be conducted such that the degree of perceived effort needed for this assessment is reduced. Furthermore, had this study been conducted in the Human Anatomy and Physiology I lab, it possibly would have eliminated the de-motivational effect of course expectation, as the students would have had less prior experience for comparison, and the benefit of the puzzle-based pedagogy may have been even more dramatically demonstrated.

## Conclusion

In keeping with numerous other studies that have highlighted the importance of pedagogical style in various courses, our findings support the critical role of pedagogy in student performance. This study also emphasizes the popular view that a broad contextual framework improves a student’s ability to recall specific details and that the individual characteristics of an instructor play a significant role in utilizing pedagogy to influence students’ performance. Additionally, our findings indicate that there is currently no assessment that can effectively quantify broader conceptual understanding across multiple fields of academia. In conclusion, a puzzle-based pedagogy, when compared to traditional lecture-based teaching, can effectively enhance the performance of students on assessments, even when the assessments only test a limited conceptual understanding of the material.
